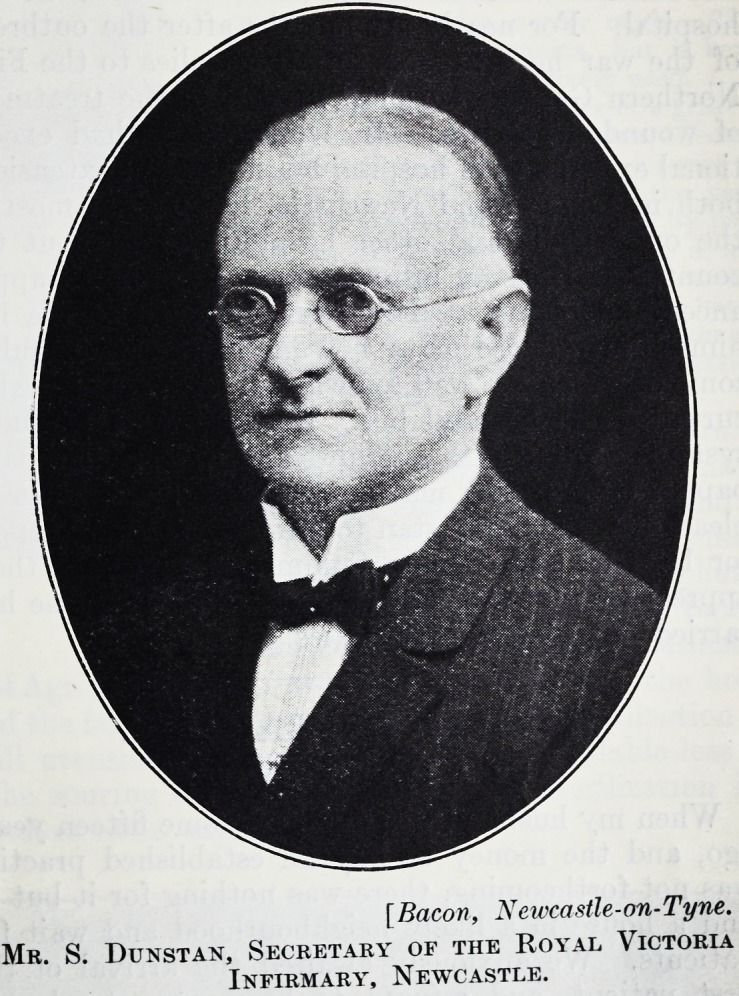# Hospital Men of Mark: Lord Armstrong and Mr. S. Dunstan

**Published:** 1924-09

**Authors:** 


### HOSPITAL MEN OF MARK.

### LORD ARMSTRONG AND MR. S. DUNSTAN.

The years 1735 to 1755 mark a period of great activity in the founding of hospitals, when the conception of a hospital erected by public subscriptions and supported by the public for "the sick and lame poor" came to be first understood. In 1735 the first provincial hospital came into being on the founding of the General Hospital at Bristol, and it was followed between that date and 1755 by some fifteen other provincial hospitals. The Newcastle Infirmary, when officially opened in 1753—the foundation-stone was laid on September 5, 1751, by Bishop Butler, of the famous "Analogy"—was found to have cost £3,679 and had accommodation for ninety patients. The first annual report, published in 1751, states that the income of the hospital was £2,643, while the expenditure amounted to £1,189—less than half the amount expended annually on milk alone at the present day, while the total annual expenditure is in the region of £100,000. The rules formulated in 1755 make amusing reading. "Patients were constantly to attend prayers" and not to "swear, curse or use abusive language." They were not to "presume to play at cards, dice or any other game," or even to smoke in the wards. No medicine was to be given to out-patients "until they deliver such vials, gallipots and such medicines as they have not taken." The apothecary was paid, including diet, washing and lodging, the sum of £30 per annum, the matron £10 and the head porter £5.

Towards the closing years of Queen Victoria's reign it was realised that the then Royal Infirmary had become totally inadequate to the needs of the district and that the building was out of date and not in a suitable position, and a public subscription was inaugurated by Sir Riley Lord when Mayor of Newcastle by which about £100,000 was raised from the public, while the late Mr. John Hall added a magnificent donation of £100,000, and Lord Armstrong subsequently added a similar amount. On June 20, 1900, the Prince of Wales laid the foundation-stone, and on July 11, 1906, as King Edward VII., accompanied by Queen Alexandra, opened the new infirmary under the title of the Royal Victoria Infirmary. The hospital stands in twenty-five acres of ground, the gift of the Freemen of Newcastle, which once formed part of the celebrated Town Moor.

[*Lafayette.*

LORD ARMSTRONG, CHAIRMAN OF THE ROYAL VICTORIA INFIRMARY, NEWCASTLE.

[*Bacon, Newcastle-on-Tyne.*

MR. S. DUNSTAN, SECRETARY OF THE ROYAL VICTORIA INFIRMARY, NEWCASTLE.

The present building has accommodation for 534 patients, but when the Orthopædic Hospital, erected in the grounds and occupied by the Ministry of Pensions is taken over, the accommodation will be raised approximately to 1,000 beds.

The Royal Victoria Infirmary is fortunate in having as its chairman one so widely experienced in hospital affairs as Lord Armstrong. He has long been a generous benefactor, is vice-president and trustee, and to commemorate his services a ward in the hospital has been named the "Armstrong Ward." He is president of many institutions in Newcastle; he founded the Barrasford Sanatorium and was its president until it was taken over by the Newcastle Corporation. He is also chairman of the North-Eastern Regional Committee of the British Hospitals Association, and takes a keen interest in all matters relating to the welfare and care of the sick. His name and his efforts on behalf of the charities of Tyneside will ever be remembered.

Mr. S. Dunstan succeeded Mr. Roden Orde as house governor and secretary in 1922. He is a native of Cornwall, and after much time devoted to the study of chemistry he accepted a position in London with one of the largest wholesale chemists in England, where for more than four years he gained much experience in the many branches of that work. He then took the position of works manager to another wholesale firm in London, and later applied himself to the study of hospital departmental work and was for many years associated with Mr. E. W. Morris, of the London Hospital. Later he was appointed to an important position at the Royal Infirmary, Newcastle-on-Tyne, being selected out of 180 applicants. Here Mr. Dunstan had exceptional opportunities of making use of his previous experiences in his responsible work of arranging and equipping several new departments, and later of the buying and distribution of all commodities used in the hospital. For nearly six months after the outbreak of the war he arranged for all supplies to the First Northern General Hospital needed in the treatment of wounded soldiers. Mr. Dunstan has had exceptional experience in hospital buildings and extensions both in London and Newcastle, has visited most of the orthopædic and other hospitals throughout the country and has an intimate knowledge of all appliances and plants used in relation thereto. He has himself introduced many new methods and formulas, some of which are well known and have considerably curtailed the hospital bill. He has also introduced systems dealing with accounts and has written many papers on hospital matters generally. It must be pleasant for Mr. Dunstan to see in the annual report for 1923 that the House Committee expressed their appreciation on the efficient manner in which he has carried out his arduous duties.

## Figures and Tables

**Figure f1:**
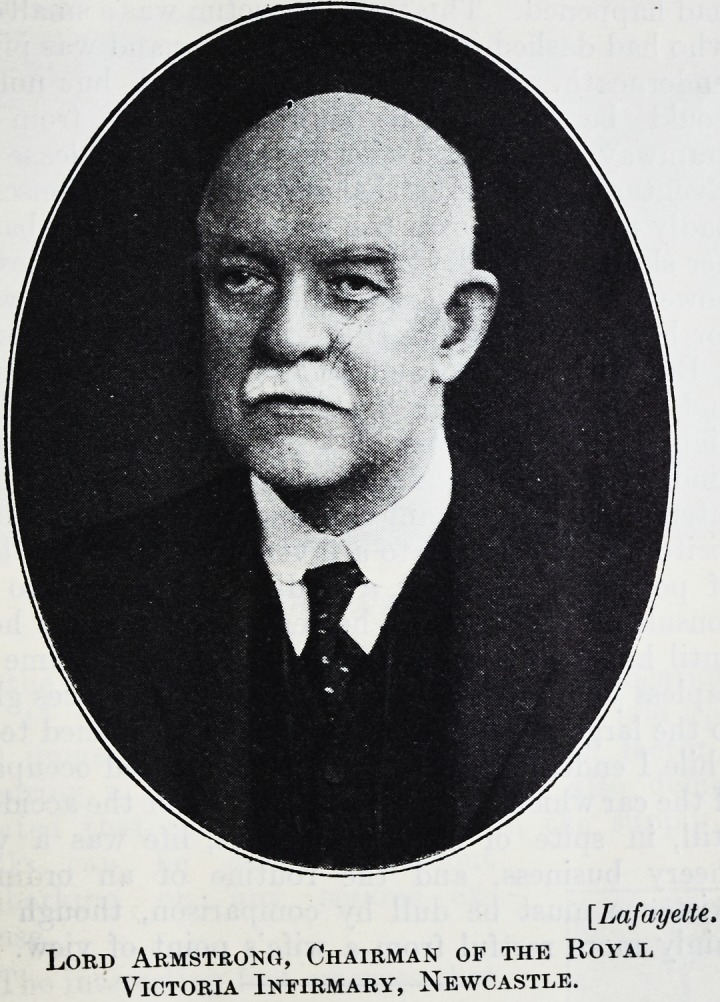


**Figure f2:**